# Impact of Digital Health on Patient-Provider Relationships in Respiratory Secondary Care Based on Qualitative and Quantitative Evidence: Systematic Review

**DOI:** 10.2196/70970

**Published:** 2025-05-30

**Authors:** Michaela Senek, David Drummond, Hilary Pinnock, Kjeld Hansen, Anshu Ankolekar, Úna O'Connor, Apolline Gonsard, Oleksandr Mazulov, Katherina Bernadette Sreter, Christina Thornton, Pippa Powell

**Affiliations:** 1 School of Medicine and Population Health Sheffield Centre for Health and Related Research (SCHARR) University of Sheffield Sheffield United Kingdom; 2 Department of Pediatric Pulmonology and Allergology University Hospital Necker Enfants Malades, APHP Université Paris Cité Paris France; 3 Usher Institute The University of Edinburgh Edinburgh United Kingdom; 4 European Lung Foundation Sheffield United Kingdom; 5 The D-Lab, Department of Precision Medicine GROW - Research Institute for Oncology and Reproduction Maastricht University Maastricht The Netherlands; 6 Department of Respiratory University Hospital Limerick HSE Mid West Limerick Ireland; 7 First Pediatric Department National Pirogov Memorial Medical University Vinnytsia Ukraine; 8 Department of Pulmonology University Hospital Centre "Sestre Milosrdnice" Zagreb Croatia; 9 Department of Medicine University of Calgary Calgary, AB Canada

**Keywords:** telemedicine, digital health, patient-provider relationship, respiratory care, systematic review, trust

## Abstract

**Background:**

Digital health technology adoption has accelerated in respiratory care, particularly since the COVID-19 pandemic, supporting various applications from self-management to telerehabilitation. While these technologies have transformed health care delivery, their impact on the patient-provider relationship in specialist respiratory care remains poorly understood.

**Objective:**

This study aims to systematically review the literature on the impact of digital health technology on the patient-provider relationship in respiratory secondary care settings and to understand the factors that enhance or diminish this relationship.

**Methods:**

In December 2023, we conducted a systematic review following Cochrane methodology, searching MEDLINE, Embase, CINAHL, Cochrane databases, and PsycINFO. We included qualitative, quantitative, and mixed methods studies examining digital health interventions in respiratory secondary care. Trained volunteers from the European Respiratory Society CONNECT Clinical Research Collaboration performed screening and data extraction. We conducted a qualitative meta-synthesis of findings, followed by an abductive quantitative data analysis. A total of 3 stakeholder workshops were held to interpret findings collaboratively with patients and health care professionals.

**Results:**

From 15,779 papers screened, 97 met the inclusion criteria (55 qualitative/mixed-methods studies, 42 quantitative studies). Studies covered various respiratory conditions, including COPD (32%), asthma (26%), and COVID-19 (13%). Four main themes emerged: trust (foundational to the relationship), adoption factors (including clinical context and implementation drivers), confidence in technology (based on functionality and the evidence base), and connection (encompassing communication and a caring presence). Digital health technology can either enhance or diminish trust between patients and clinicians, with patients' perceptions of the motivations behind its implementation being crucial. While technology facilitated access and communication, remote consultations risked depersonalisation, particularly when not balanced with in-person interactions. Self-monitoring and access to information empowered patients and promoted more equitable patient-provider relationships.

**Conclusions:**

Digital health technology can either strengthen or weaken patient-provider relationships in respiratory care, with effects impacted by adoption factors, confidence in technology, connection, and patient empowerment. Maintaining trust in the era of digital care requires transparent implementation of motivations, consideration of individual circumstances, and reliable technology that supports rather than replaces the therapeutic relationship.

**Trial Registration:**

PROSPERO CRD42024493664; https://www.crd.york.ac.uk/PROSPERO/view/CRD42024493664

## Introduction

### Digital Health Technologies and Their Benefits

Digital technologies encompass a broad range of tools, including telemedicine, electronic health records, mobile health apps, wearable devices, and artificial intelligence (AI)–assisted diagnostics [1]. These technologies have significantly transformed health care delivery by improving accessibility, efficiency, and continuity of care. The World Health Organization (WHO) has highlighted digital health as a strategy to achieve universal health care coverage, particularly by facilitating remote care and enhancing support for vulnerable populations [2,3]. Digital health tools also allow for remote monitoring of disease progression, support for assisted living, and implementation of cloud-based health care systems.

### Acceleration of Digital Health Adoption During the COVID-19 Pandemic

The adoption of digital health technologies accelerated dramatically during the COVID-19 pandemic as health care systems adapted to the need for remote care. The crisis led to a surge in teleconsultations, virtual wards, home treatments, and digital self-management tools, many of which have persisted beyond the pandemic [4]. Both patients and health care providers embraced these changes, recognizing their potential to enhance access to care while reducing unnecessary hospital visits. However, the rapid shift to digital health care also raised concerns about digital equity, access disparities, and the potential depersonalization of care.

### Relevance for Patients With Respiratory Disease

Patients with respiratory diseases were among the groups most affected by the digital transformation of health care [5]. Conditions such as chronic obstructive pulmonary disease (COPD), asthma, and cystic fibrosis require ongoing monitoring and frequent interactions with health care providers. Given the complexity of managing respiratory diseases, the shift to digital care also raised concerns regarding patient engagement, adherence to treatment, and the impact on the patient-provider relationship, particularly in the secondary care setting, where interactions are typically episodic rather than continuous.

### Impact of Digital Health on the Patient-Provider Relationship

Predating widespread adoption of digital health, Ridd et al [6] in primary care conceptualized the patient-provider relationship as being built on continuity, interpersonal communication, and trust. Their framework emphasized that long-term patient-provider interactions foster deeper connections, leading to better health outcomes.

In the digital era, the dynamics of these relationships have shifted. In a review of reviews, Ramachandran et al [7] found that eHealth technologies have a mixed impact on relationships and trust in primary care patient-provider interactions, depending on patient perceptions, provider communication skills, technology design, and organizational factors. They concluded that training providers in technology-specific communication skills and ensuring that eHealth implementations were equitable and considerate of diverse patient needs can foster trust and maintain strong relationships. Similarly, a scoping review that explored the theoretical perspectives underpinning research on the physician-patient relationship in digital health practice emphasized the need for ethical considerations in medical practice to ensure that technology enhances, rather than hinders, the quality of care [8].

These reviews have focused on primary care, where patients benefit from long-term relationships with a single local provider. In contrast, in secondary care, interactions may be with multiple specialists sometimes at a substantial distance, typically short-term rather than continuous. This was a key theme raised by patients and professionals within the Institute for Healthcare Improvement-funded Rapid and Secure AI-enhanced Diagnosis, Precision Medicine and Patient Empowerment Centered Decision Support System for Coronavirus Pandemics (DRAGON) project, which focused on care during the COVID-19 pandemic. We, therefore, aimed to systematically review the literature on the impact of digital health technology on the patient-provider relationship and to understand the factors that enhance or diminish it in respiratory secondary care settings.

## Methods

### Overview

Our systematic review is registered with the International Prospective Register of Systematic Reviews (PROSPERO; CRD42024493664) and follows the Cochrane methodology [9] and the PRISMA (Preferred Reporting Items for Systematic Reviews and Meta-Analyses) guidelines. There were no deviations from the registered protocol. We used Covidence software to manage the screening and data extraction process [10].

### Search Strategy and Selection Criteria

The search strategy had three components: digital health care, respiratory conditions (including COVID-19), and patient-provider relationships. Detailed methods are outlined in Multimedia Appendix 1. We applied English-language search filters but no study design or date limits. Searches were conducted in December 2023 in Ovid MEDLINE, Embase, and CINAHL via ESBCO, Cochrane Database of Systematic Reviews, Cochrane Central Register of Controlled Trials, and PsycINFO. Duplicates were detected and removed using Covidence software.

#### Eligibility Criteria

Our inclusion and exclusion criteria are presented in Textbox 1.

**Inclusion and exclusion criteria.**

**Inclusion criteria**

- Population: respiratory disease patients, both adult and pediatric.
- Interventions: eHealth technology, mobile health technology, or artificial intelligence.
- Comparator: standard care (if appropriate: eg, in controlled intervention studies)
- Outcome: the quality of the patient-provider relationship changes in the patient-provider relationship.
- Study design: qualitative, mixed-methods studies, randomized controlled trials, cross-sectional
- Setting: secondary care settings only. This includes hospital or clinic-based medical specialist care and can consist of urgent and emergency care, or planned elective care.

**Exclusion criteria**

- Systematic reviews, abstracts, conference papers, protocols or commentaries, and studies in primary care settings.

#### Screening and Data Extraction

Following a training program, 30 volunteer clinicians and researchers from the European Respiratory Society CONNECT Clinical Research Collaboration [11] contributed to the screening of titles and abstracts. The volunteers came from various health care professions, including doctors, nurses, physiotherapists, clinicians, academic researchers, and basic scientists. The training program consisted of multiple stages. For screening titles and abstracts, reviewers attended a session where they received detailed screening guidelines, including clear criteria for “yes,” “no,” and “maybe” decisions. Articles meeting all 3 key criteria (digital health, patient-provider relationship, and respiratory disease) were marked as “yes.” Articles covering none or only one of these areas were marked as “no.” Any articles where reviewers were uncertain or partially met criteria were marked as “maybe.” Following this initial training, reviewers completed a practical exercise screening 100 test articles to gain hands-on experience with the criteria. After 2 weeks of independent screening practice, a second session was held to address questions that had emerged during the preliminary screening exercise and clarify any areas of uncertainty. All articles marked as “maybe” were subsequently reviewed by 2 senior reviewers to ensure quality and consistency. All authors and DRAGON or CONNECT group members performed the title and abstract stage. A total of 6 reviewers (MS, DD, HP, PP, AK, and KH) led by MS and DD resolved conflicts and oversaw quality [12]. Following a similar multistage training program, MS, DD, PP, HP, KH, OM, AA, CT, AG, KBS, and UC conducted the full-text screening with MS and DD, resolving conflicts and overseeing quality.

#### Quality Assessment

A total of 2 reviewers assessed the methodological quality of the studies using the relevant Critical Appraisal Skills Programme (CASP) checklist [8]. We selected this tool because it includes an assessment of the paper’s relevance to the review aim (“Will the results help our review?”). We anticipated that the patient-provider relationship was unlikely to be a primary objective in most studies, so using the CASP tool enabled us to identify and emphasize the findings from studies whose aims aligned most closely with our research question. CASP does not generate a total score. We classified the studies as those that explored the patient-provider relationship: (1) as the primary aim, (2) as a secondary objective when the authors had explicitly defined it as such in the methods section, or (3) as a coincidental outcome when it was identified in the results but not prespecified in the methods.

### Data Analysis

#### Qualitative Meta-Synthesis

We initially applied the framework from Ridd et al [6], which defines knowledge, trust, loyalty, and regard as constructs of the depth of a patient-provider relationship within the long-term context of primary care relationships. However, because it did not reflect all aspects of digital health care as described in our included papers, MS and DD undertook a thematic meta-synthesis. The data were coded into subthemes and overarching themes using Quirkos software (version 2.3).

#### Abductive Analysis for Quantitative and Mixed Methods Findings

Categories emerging from the qualitative meta-synthesis provided a framework to organize the quantitative data. A total of 2 researchers (MS and DD) followed an abduction process [13] to consider narratives around elements of the patient-provider relationship affected (positively or negatively) by digital health care. We integrated objective measures (quantitative surface) and latent phenomena to understand these elements.

#### Stakeholder Engagement Workshops to Interpret Findings

Our work followed the consultation and application approaches to collaborative analysis defined by Jennings et al [14]. Aligned with the methodology of collaborative analysis [15] we presented our preliminary analysis in 3 workshops with patients, patient representatives, and health care professionals. Following an initial analysis within the research team, we carried out the workshops (one for health care professionals and managers, one for patients and patient representatives, and a final workshop for all stakeholder groups. Participants provided verbal and written feedback using Groupmap [16] and engaged in consensus activities, such as using WordCloud functions [17] to inform the interpretation of the findings. Interactive techniques (Wordclouds, GroupMap, and Chat) facilitated engagement in consensus activities, which we used to inform further analysis and interpretation of the findings.

The final analysis stage involved fully integrating the quantitative and qualitative datasets. The research team’s iterative discussions achieved this by aligning the 2 datasets and comparing what is familiar with what is unfamiliar to generate robust explanations [13].

## Results

### Overview

Following deduplication, we screened titles and abstracts of 15,779 papers; 545 were retrieved for full-text review. We included studies that reported using digital health technologies, including diagnosis, consultations, treatment, and monitoring. We included all studies that comprised respiratory disease patient groups of any age, gender, and disease severity. We excluded studies that did not examine respiratory conditions in secondary care settings or report on the patient-provider relationship.

**Figure 1:**
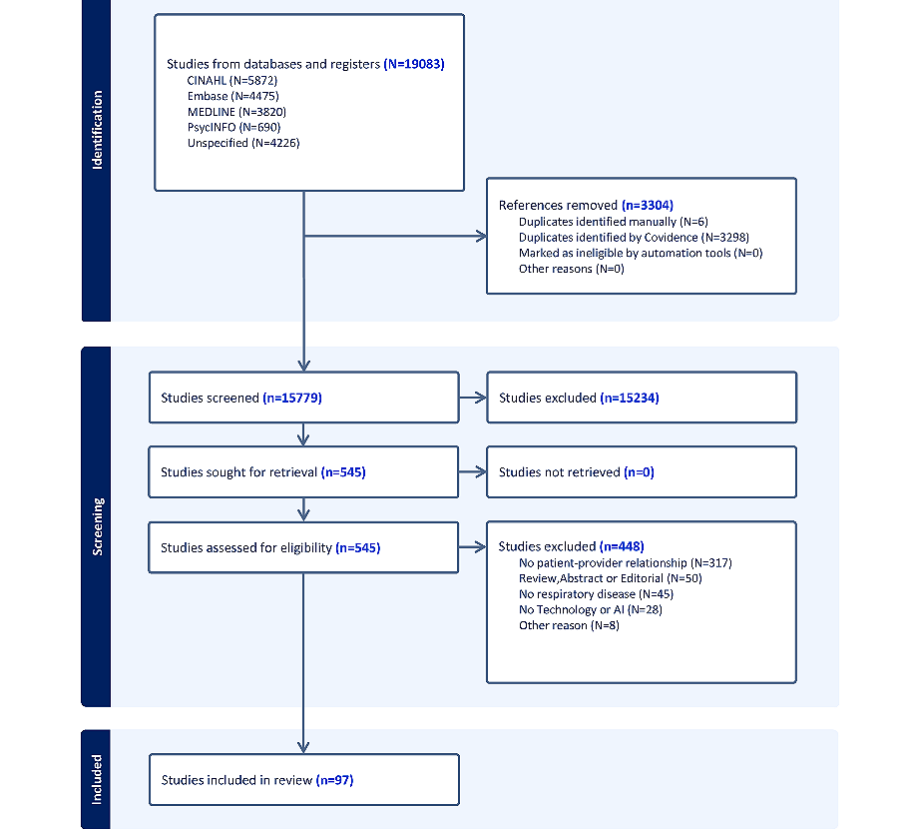
PRISMA (Preferred Reporting Items for Systematic Reviews and Meta-Analyses) flow diagram.

The primary reason for exclusion at the title and abstract screening stage was the lack of reported primary outcome of interest and the relationship between patient and provider, resulting in 15,234 excluded studies. In the second screening stage of full texts, the main reasons for exclusion were insufficient reporting on the patient-provider aspect (316 full texts excluded), patients with no respiratory disease (45 studies), wrong study design (50 studies in total), and no technology (28 studies). In total, we identified 97 studies that met our inclusion criteria and had sufficiently reported on the aspects of patient-provider relationships (see the PRISMA flow diagram in Figure 1 and checklist in Multimedia Appendix 2).

### Characteristics of the Studies Included

Tables 1 (for qualitative studies, 56/97, 58%) and 2 (for quantitative studies, 41/97, 42%) present the included studies.

A third of the studies (29/97, 30%) were carried out after the onset of the pandemic. The studies included a broad range of respiratory conditions: COPD (23/97, 24%), asthma (22/97, 23%), lung cancer (10/97, 10%), COVID-19 (9/97, 9%), tuberculosis (9/97, 9%), cystic fibrosis (7/97, 7%), sleep apnea (2/97, 2%), and interstitial lung disease (1/97, 1%) studies. The remaining 14 out of 97 (14%) studies included multiple respiratory conditions (including COVID-19). The primary aim of almost all the studies was testing a device. Only 6 out of 97 (6%) studies had a primary aim directly related to the patient-provider relationship; in 8 out of 97 (8%) studies, the relationship was an explicit secondary objective. Most studies (83/97, 86%) coincidentally reported an outcome relevant to the patient-provider relationship.

**Table 1:** Included qualitative and mixed methods studies.

Study characteristics							Theme			
Study	Applicability	Country	System type	Disease	COVID-19 era	Adoption factors		Confidence in technology	Connection	Patient empowerment
Mantzounaris^a^ [18]	Low	Greece	Telehealth (teleconsultation)	Asthma	Pre	Ctxt^b^		—^c^	Communication	Self-efficacy
Hibbert et al [19]	Moderate	United Kingdom	Telehealth (telemonitoring)	COPD^d^	Pre	—		TP^e^	Depersonalization	—
van Baar et al [20]	Moderate	United Kingdom	Telehealth (teleconsultation)	Asthma	Pre	—		—	Communication, Depersonalization	—
Whitten and Mickus [21]	Moderate	United States	Telehealth (teleconsultation)	COPD	Pre	—		—	Communication	—
Cornwall et al [22]	Moderate	United Kingdom	Communication	Lung cancer	Pre	PoM^f^		—	Communication	—
Mair et al [23]	Moderate	United Kingdom	Telehealth (telemonitoring)	COPD	Pre	Ctxt, PoM		TP, SoE^g^	CP^h^, Depersonalization	—
Hoffman et al [24]	Low	Kenya	Telehealth (telemonitoring)	Tuberculosis	Pre	—		—	Communication	—
Shany et al [25]	Low	Australia	Telehealth (telemonitoring)	COPD	Pre	—		TP	—	Self-efficacy
Cox et al [26]	Low	Australia	Telehealth (telemonitoring)	Lung cancer	Pre	Ctxt		IA^i^	Depersonalization	—
Fairbrother et al [27]	High	United Kingdom	Telehealth (telemonitoring)	COPD	Pre	—		TP	Communication, CP, CoC^j^	—
Kim et al [28]	Low	South Korea	Telehealth (telemonitoring)	COPD	Pre	—		—	Communication, CoC	—
Dinesen et al [29]	Moderate	Denmark	Telehealth (telerehabilitation)	COPD	Pre	Ctxt			CP	Self-efficacy
Huniche et al [30]	Moderate	Denmark	Telehealth (self-monitoring)	COPD	Pre	—		—	CP	Self-efficacy
Damhus et al [31]	Moderate	Denmark	Telehealth (telemonitoring)	COPD	Pre	—		—	CP	Self-efficacy
Brown-Johnson et al [32]	Low	United States	Education	Lung cancer	Pre	—		—	Communication	Self-efficacy
Kenealy et al [33]	Moderate	New Zealand	Telehealth (telemonitoring)	COPD	Pre	—		—	Communication, CP	Self-efficacy
Maguire et al [34]	Low	United Kingdom	Telehealth (telemonitoring)	Lung cancer	Pre	—		IA	Communication, CP	Self-efficacy, SDM^k^
Roberts et al [35]	Low	United States	Telehealth (teleconsultation)	Asthma	Pre	PoM		—	Communication	Self-efficacy
Dichmann Sorknaes [36]	Low	Denmark	Telehealth (teleconsultation)	COPD	Pre	—		IA	Communication, CP	—
Daftary et al [37]	Moderate	Ethiopia	Communication	Tuberculosis	Pre	Ctxt		TP, SoE	—	—
Hirsch-Moverman et al [38]	Moderate	Lesotho	Communication	Tuberculosis	Pre	PoM		—	Communication, CoC	—
Kopanitsa [39]	Moderate	Russia	Communication	Tuberculosis	Pre	PoM, DoI^l^		SoE	Communication	Self-efficacy
Nhavoto et al [40]	Moderate	Mozambique	Communication	Tuberculosis	Pre	Ctxt		—	Communication	Self-efficacy
Nissen and Lindhardt [41]	Moderate	Denmark	Telehealth (teleconsultation)	COPD	Pre	PoM		TP	Communication, CP, CoC	Self-efficacy
Damhus et al [31]	Moderate	Denmark	Telehealth (telerehabilitation)	COPD	Pre	PoM		TP, IA, SoE	Communication	—
Liacos [42] et al	Low	Australia	Telehealth (telerehabilitation)	Mixed	Pre	—		—	Communication, CP	—
Hamilton et al [43]	Moderate	United States	Decision support	Lung cancer	Pre	PoM		—	Communication	SDM
Patel et al [44]	High	United States	EHR^m^	Asthma	Pre	—		—	Communication	—
Rudin et al [45]	Moderate	United States	Telehealth (telemonitoring)	Asthma	Pre	—		—	CP	Self-efficacy
Boström et al [46]	High	Sweden	Telehealth (teleconsultation)	COPD	Pre	—		—	Communication, CP	Self-efficacy
Drabble et al [47]	Moderate	United Kingdom	Telehealth (telemonitoring)	CF^n^	Pre	—		—	CP	Self-efficacy
Kuntz et al [48]	Low	United States	Telehealth (teleconsultation)	COVID-19	Post	Ctxt, PoM		—	Communication, CP	—
Lewis et al [49]	Low	United Kingdom	Telehealth (teleconsultation)	COVID-19	Post	Ctxt		—	—	—
Maguire et al [50]	Moderate	United Kingdom	Telehealth (telemonitoring)	Lung cancer	Pre	CC		—	Communication, CP	—
Van Citters^a^ et al [51]	Moderate	United States	Telehealth (telemonitoring)	CF	Pre	PoM		—	C P	Self-efficacy, SDM
van Lieshout et al [52]	Moderate	Canada	Telehealth (telemonitoring)	COPD	Pre	Ctxt, PoM, DoI		TP, SoE	Communication, CoC	Self-efficacy
Yamada et al [53]	Low	Canada	Decision support	Asthma	Pre	Ctxt, PoM		—	—	Self-efficacy, SDM
Bains et al^a^ [54]	Low	United States	Telehealth (teleconsultation)	COVID-19	Post	—		—	Communication, CP	—
Jácome et al [55]	Low	Portugal	Telehealth (teleconsultation)	Asthma	Pre	—		—	Communication, CoC	Self-efficacy
Kennedy et al [56]	High	United States	Telehealth (teleconsultation)	Mixed	Post	Ctxt		TP	Communication, Depersonalization	—
Legler et al [57]	Moderate	United States	Telehealth (teleconsultation)	COVID-19	Post	PoM		—	Communication, CP, Depersonalization	—
Sekandi et al [58]	Moderate	Uganda	Telehealth (telemonitoring)	Tuberculosis	Pre	Ctxt, PoM		IA	Communication, CP, CoC	—
Thomas et al [59]	Moderate	India	Telehealth (telemonitoring)	Tuberculosis	Pre	PoM		—	CP	—
Wu et al [60]	Low	United Kingdom	Telehealth (teleconsultation)	COPD	Post	Ctxt, PoM		—	—	Self-efficacy, SDM
Gillett and Hope-Gill [61]	Moderate	United Kingdom	Telehealth (teleconsultation)	ILD^o^	Post	PoM, CC		IA	Depersonalization	—
Haynes^a^ et al [62]	Moderate	United States	Telehealth (teleconsultation)	Asthma	Post	Ctxt, PoM		—	Communication, Depersonalization	—
Jiang et al [63]	Moderate	China	Telehealth (telerehabilitation)	COPD	Post	Ctxt, PoM		IA, SoE	Depersonalization	Self-efficacy
Makhecha et al [64]	Low	United Kingdom	Telehealth (teleconsultation)	Mixed	Post	—		—	Communication, Depersonalization	—
Misplon et al [65]	Low	Belgium	Telehealth (telemonitoring)	Lung Cancer	Post	—		—	Communication	—
Nayyar et al [66]	Moderate	Canada	Telehealth (teleconsultation)	Mixed	Post	Ctxt, PoM		—	Depersonalization	—
Sandau et al [67]	Low	Denmark	Medical device	COPD	Post	PoM		TP	CP, Depersonalization	—
Cox et al [68]	Low	New Zealand	Telehealth (telerehabilitation)	Mixed	Pre	Ctxt		TP	Depersonalization	Self-efficacy, SDM
Hattingh et al [69]	Moderate	Australia	Telehealth (teleconsultation)	COVID-19	Post	Ctxt			Communication, CoC	
Kazmerski et al [70]	Moderate	United States	Telehealth (teleconsultation)	CF	Post	—		IA	—	Self-efficacy, SDM
Robinson et al [71]	Low	United Kingdom	Diagnostics	COPD	Post	—		TP, IA, SoE	CP, CoC	Self-efficacy
Watanabe et al [72]	Moderate	United States	Telehealth (self-monitoring)	CF	Pre	—		—	Communication	Self-efficacy, SDM

^a^Studies that included children.

^b^Ctxt: context.

^c^Not applicable.

^d^COPD: chronic obstructive pulmonary disease.

^e^TP: technology performance.

^f^PoM: perception of motivations.

^g^SoE: strength of evidence.

^h^CP: caring presence.

^i^IA: interaction anxiety.

^j^CoC: continuity of care.

^k^SDM: shared decision-making.

^l^DoI: drivers of implementation.

^m^EHR: electronic health record.

^n^CF: cystic fibrosis.

^o^ILD: interstitial lung disease.

**Table 2:** Included quantitative studies.

Study characteristics							Theme			
Study	Applicability	Country	System type	Disease	COVID-19 era	Adoption factors		Confidence in technology	Connection	Patient empowerment
Pacht et al [73]	Moderate	United States	Telehealth (teleconsultation)	Mix of respiratory diseases	Pre	—^a^		—	Communication	Self-efficacy
Stepnowsky et al [74]	Moderate	United States	Telehealth (telemonitoring)	Sleep apnea	Pre	—		—	Communication, Depersonalization, CoC^b^	—
Varkey et al [75]	Moderate	United States	Telehealth (telemonitoring)	Mix of respiratory diseases	Pre	PoM^c^		TP^d^, SoE^e^	Communication	—
Agha et al [76]	Moderate	United States	Telehealth (teleconsultation)	Mix of respiratory diseases	Pre	—		—	Communication, Depersonalization	—
Zamith et al [77]	Low	Portugal	Telehealth (Teleconsultation)	Mix of respiratory diseases	Pre	CoC, PoM		—	CP^f^	—
Byczkowsi et al^g^ [78]	Low	United States	Telehealth (Telemonitoring)	CF^h^	Pre	—		IA^i^	Communication, Depersonalization	—
Nield and Hoo [79]	Low	United States	Telehealth (Teleconsultation)	COPD^j^	Pre	—		—	CP	Self-efficacy
Chih et al [80]	Low	United States	Telehealth (Teleconsultation)	Lung cancer	Pre	—		—	Communication, CP	Self-efficacy
Pinnock et al [81]	Low	United Kingdom	Telehealth (Telemonitoring)	COPD	Pre	—		—	CoC	Self-efficacy
Cingi et al [82]	Low	Turkey	Telehealth (Teleconsultation)	Asthma	Pre	—		TP, SoE	Communication, CP	—
Apter et al [83]	Low	United States	Telehealth (Teleconsultation)	Asthma	Pre	PoM, CoC		—	Communication, CP	—
Wiecha^g^ et al [84]	Low	United States	Telehealth (teleconsultation)	Asthma	Pre	PoM		—	Communication, CP	Self-efficacy
Fadaizadeh et al [85]	Moderate	Iran	Telehealth (Telemonitoring)	Asthma	Pre	—		TP, IA	Communication, CP	—
Rosenberger et al [86]	Low	United States	Telehealth (Telemonitoring)	Mix	Pre	DoI^k^		TP	Communication, CP	—
Poureslami [87]	Low	Canada	Telehealth (Teleconsultation)	Asthma	Pre	—		IA	Depersonalization	Self-efficacy
Sleath^g^ et al [88]	High	United States	Telehealth (teleconsultation)	Asthma	Pre	DoI		—	Communication, CP	Self-efficacy
Morita et al [89]	Low	Canada	Telehealth (telemonitoring)	Asthma	Pre	—		—	Communication, Depersonalization	Self-efficacy,
Sonney^g^ et al [90]	Low	United States	Telehealth (telemonitoring)	Asthma	Pre	—		TP	Communication	Self-efficacy, SDM^l^
Apter et al [91]	Low	United States	Communication	Asthma	Pre	—		IA	Communication, CP	Self-efficacy
Gaynor et al [92]	Low	United States	Telehealth (teleconsultation)	Asthma	Pre	DoI		—	Communication	Self-efficacy, SDM
Kneuertz et al [93]	Low	United States	Telehealth (teleconsultation)	Lung cancer	Pre	—		—	CoC, CP	Self-efficacy
Koff et al [94]	Low	United States	Telehealth (telemonitoring)	COPD	Pre	—		—	—	Self-efficacy, SDM
Opipari-Arrigan^g^ et al [95]	Moderate	United States	Communication	CF	Pre	—		—	Communication	Self-efficacy, SDM
Perlman et al [96]	Low	United States	Telehealth (teleconsultation)	COVID-19	Post	PoM		—	Communication	Self-efficacy, SDM
Al-Sharif [97]	Low	Saudi Arabia	Telehealth (telemonitoring)	COPD	Post	—		IA, SoE	Communication, Depersonalization	—
Davis^g^ [98]	Low	United States	Telehealth (teleconsultation)	Mix of respiratory diseases	Post	PoM		IA, SoE	Depersonalization	—
Gashu et al [99]	Moderate	Ethiopia	Telehealth (teleconsultation)	Tuberculosis	Post	—		—	Communication, CP	Self-efficacy
Hendra et al [100]	Moderate	United States	Telehealth (teleconsultation)	CF	Pre	CoC		IA	—	Self-efficacy
Meshkov et al [101]	Low	Russia	Communication	Tuberculosis	Pre	—		—	Communication	—
Kowatsch^g^ et al [102]	Low	Switzerland	Telehealth (teleconsultation)	Asthma	Pre	PoM		—	Communication, Depersonalization	—
Mustafa et al [103]	Low	United States	Telehealth (teleconsultation)	Asthma	Post	Ctxt^m^		—	Depersonalization	—
Sousa [104]	Moderate	Portugal	Telehealth (teleconsultation)	Asthma	Post	Ctxt		—	Communication, Depersonalization	—
Benson et al [105]	Low	United States	Telehealth (teleconsultation)	Lung cancer	Pre	CoC		IA	Depersonalization	—
Bukstein et al [106]	Low	United States	Telehealth (teleconsultation)	Mix of respiratory diseases	Post	—		SoE, IA	Depersonalization	—
Nelson et al [107]	Low	United States	Telehealth (teleconsultation)	Mixed	Post	Ctxt		—	Communication, Depersonalization	—
Shinoda et al [108]	Moderate	Japan	Telehealth (telemonitoring)	Mix of respiratory diseases	Post	PoM		—	Depersonalization	Self-efficacy
Chun et al [109]	Low	United States	Telehealth (telemonitoring)	Sleep apnea	Pre	—		TP, SoE	Communication, CoC	—
Coker^g^ et al [110]	Moderate	United States	Telehealth (teleconsultation)	Asthma	Post	—		—	Communication	Self-efficacy, SDM
Lawrence^g^ et al [111]	Low	Australia	Telehealth (teleconsultation)	COVID-19	Post	PoM		—	Communication, CoC	—
Varghese et al [112]	High	India	Telehealth (teleconsultation)	COVID-19	Post	—		—	Communication	—
Zhuge et al [113]	Moderate	China	Diagnostics	COVID-19	Post	Ctxt, PoM		—	Communication	—

^a^Not applicable.

^b^CoC: continuity of care.

^c^PoM: perception of motivations.

^d^TP: technology performance.

^e^SoE: strength of evidence.

^f^CP: caring presence.

^g^Studies which included children.

^h^CF: Cystic fibrosis.

^i^IA: interaction anxiety.

^j^COPD: chronic obstructive pulmonary disease.

^k^DoI: drivers of implementation.

^l^SDM: shared decision making.

^m^Ctxt: context.

### Themes from the Integrative Analysis

**Figure 2:**
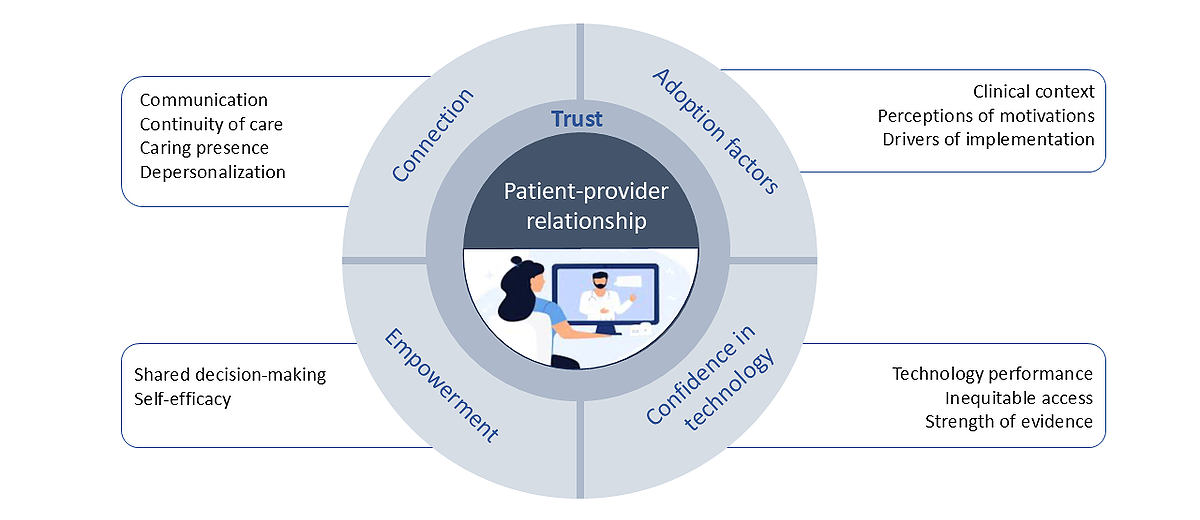
Themes and subthemes.

Initial coding resulted in 12 subthemes synthesized into four main themes: adoption factors, confidence in technology, connection, and empowerment (Figure 2). The figure illustrates how each of the four main themes (each described below) influences the trust within the patient-provider relationship. The quantitative data collected corroborated our 4 main themes. Detailed examples of supporting quantitative evidence for each theme and subtheme are presented in Multimedia Appendix 3, strengthening our thematic framework through methodological triangulation.

Figure 2 should be interpreted from the outer elements inwards, illustrating how each layer influences the next: digital respiratory care affects intermediate themes (eg, continuity of care), which influence core themes (eg, connection), ultimately impacting trust within the patient-provider relationship at the center.

We describe the four themes below using data extracts to clarify and confirm their meanings. Each theme was reasonably equally weighted in frequency of occurrence.

#### Trust

Trust emerged as the foundational element in the patient-provider relationship. Trust was defined as “the patient’s confidence that the physician will do what is best for the patient” [6]. Digital care could impact the other four themes (adoption factors, confidence in technology, connection, and empowerment), enhancing or diminishing trust.

#### Adoption Factors

The clinical context significantly influenced the adoption [61]. In the context of a pandemic, where visiting hospitals was unsafe, remote care was more acceptable [114].

I think it’s (telephone consulting) the norm because of the circumstances…nobody wants to get COVID [61].

telephone consultations during the pandemic

The severity of the patient’s condition also affected their confidence in using digital health technologies. Those with milder conditions were more willing to use them compared to those with severe or complex conditions, who may prefer in-person consultations [106], for instance, older patients with respiratory diseases during the pandemic).

Patients’ perceptions of the drivers of implementation and the motivation behind using digital health technologies influenced trust. Policy, organizations, and clinicians could drive technology implementation. Or it could be a complex sequence of these as policy pushed organizations to implement change, which then pushed professionals to use technology [115]. Patients could also advocate for using digital health technologies [71].

Patients’ trust was enhanced when digital health care was implemented to increase patient safety and convenience and improve outcomes [100], and it was diminished if technology was used primarily to save time and resources [116].

#### Confidence in Technology

Trust in technology was intertwined with trust in the health care providers who recommended the technology [117], shaping the overall connection and trust built within the relationship [118]. Patients’ trust in clinicians’ professional judgment was interrelated with how trustworthy patients perceived the technology [40].

Confidence in technology is the belief that you can use technology with ease and competence and that it is reliable. Digital health technology’s performance affects confidence in its functionality. Technology that is not intuitive or functions poorly leads to a loss of confidence, negatively impacting the patient-provider relationship.

Due to technical difficulties, data went missing….There was some loss of trust in the system, and some patients had more outpatient visits and hospital visits [33].

Kenealy et al, 2015

There was a risk of increasing inequities as socioeconomic and demographic factors affected confidence and trust in the technology. For some populations, such as older adults [37], or socioeconomically deprived, accessing health care professionals through technology was challenging.

So we speed ahead with the technology. We think this is all great, but many people do not have access to the right environment. How do you have a confidential conversation with somebody living in a one-bedroom apartment with four people? [66]

Nayyar et al, 2022

For health care providers, the strength of the evidence supporting its use and reliability in clinical practice affected confidence in technology. Clinicians reported instances where technology suggested additional treatment was needed despite their clinical judgment indicating otherwise. In addition, providers were concerned that some patients placed excessive trust in health technology that could prove unreliable [43].

#### Connection

On a practical level, technology improved patient and professional access and communication:

If I need anything, I have just to phone up…it is a good service [27].

COPD telemonitoring, prepandemic

Continuity of care using telehealth technologies enhanced connection and trust [41] and improved treatment adherence [40]. Clinicians valued continuity of care, observing that patients were more trusting when the same provider consistently performed telemonitoring [45].

I think it is probably best if [telemonitoring is done by] people that are dealing with the patients every single day and have that bit of a relationship with them ... people are more trusting, and it is something more personable for the patient [27].

COPD telemonitoring, prepandemic

Technology can reassure patients that someone is “watching over them” and create a feeling of a “caring presence” compensating for depersonalization due to a lack of physical presence [71].

Grateful that someone kept an eagle eye on my son as it was a very stressful time given his other health issues. Stress levels were significantly reduced by being in his home environment and knowing [the doctor] was a video call away, and he was being monitored remotely. [71]

Physical activity intervention for COPD

However, both patients and providers reported depersonalization due to a lack of in-person interactions, and both expressed a preference for a combination of in-person and remote consultations: In-person consultations could form a stronger bond and strengthen the relationship.

A physical meeting creates more opportunities to instil security and peace. It has an inherent energy, creating a connection, where patients and relatives can ask questions and be recognised. [113]

COPD management in the Swedish population

A study by Jiang et al [63] further emphasized the sense of depersonalization that may occur due to the physical distance during remote consultations.

It is not just the exchange of disease information ... eye contact and a gesture from a doctor or a nurse will give the patient much psychological comfort, which is very difficult to achieve on a mobile phone. [63]

COPD management study for older patients

Both clinicians and patients found remote interactions with a new provider difficult and highlighted that it was difficult to establish trust and rapport with a new provider using telemedicine [62].

#### Empowerment

Empowerment is important for a balanced patient-provider relationship. Empowered patients have more control and are often involved in decisions about their care, such as how and where services are delivered. Technology helped patients be better informed about their condition [65] and increased their self-efficacy and confidence through home-collected data [33]. This enabled active participation in self-management, enhanced self-efficacy, supported shared decision-making, and facilitated a more equal patient-provider relationship.

Patients feel empowered when contacting the clinician because they have vital readings and their sense of health to explain how they feel and get support. [29]

COPD self-monitoring, prepandemic

#### Stakeholder Workshops and Interpretation of Findings

Our work followed the consultation and application approaches to collaborative analysis [14]. Following data analysis, and once we had preliminary findings, we carried out stakeholder engagement sessions, where we presented initial findings from the review to stakeholders for feedback. The sessions were conducted remotely and were interactive. All participants were asked to provide verbal and written feedback using Groupmap, WordCloud, and group chat.

The discussion among clinicians and patients at the stakeholder workshops resonated with our findings. The word associations related to digital health technology collected in WordCloud at the start of the session generally reflected positive features of technology identified in our analysis (accessibility, convenience, modernization, and education). At the end of the meeting, after considerable discussion, views were more nuanced, and some of the associations were neutral or more negative (accessibility, followed by disparities, frustrating, potential, complex, and challenge). These views increased confidence in our conclusions as the very positive interpretations from some papers were tempered by the more cautious considerations highlighted in other studies.

## Discussion

### Principal Findings

We have synthesized the findings of 97 papers on the impact of digital health technology on the patient-professional relationship in specialist practice. Both qualitative and quantitative evidence were used to identify the themes and subthemes, with the qualitative findings revealing underlying nuances and contextual factors. This approach enabled us to quantify key patterns in technology adoption, confidence, connection, and empowerment, while simultaneously understanding the mechanisms through which these factors influenced the patient-provider relationship. Trust was the foundational theme, and digital health technology could either enhance or reduce trust between patients and clinicians. Patients’ perception of why digital health technology was implemented (*eg,* whether it was used to benefit patient care or used to save time and resources) was a significant factor. The technology could connect patients and clinicians by facilitating access, communication, and continuity of care, but remote consultations risk depersonalization, especially if they are not balanced with in-person interactions. Self-monitoring and access to information can empower patients and promote a more equal patient-provider relationship. Combining qualitative and quantitative measures has provided a fuller picture of patient-provider interactions, as each method offers unique insights into different aspects of the relationship. Qualitative insights into how technology empowers patients to seek information and engage in health care decisions are supported by quantitative data showing improved service engagement and compliance. Integrating qualitative and quantitative data has enriched our understanding of patient-provider relationships by linking communication styles and patient engagement with measurable outcomes. These findings complement each other and can lead to more effective healthcare strategies and improved patient care.

### The Pivotal Role of Trust

Trust in the patient-professional relationship was intertwined with trust in technology. While Ridd et al [6] identified trust as a critical factor in determining the depth of the patient and professional relationship in their 2009 primary care study, digital health care technology has introduced a third actor, which can positively or negatively influence relationship formation, development, and maintenance.

Patients’ trust in technology was fundamentally linked to their trust in clinicians. We found that patients trusted technology when they trusted their clinicians to provide the best care and achieve desired outcomes, reflecting broader evidence that patients build trust through receiving quality care in well-functioning health systems [119]. When technology or systems fail, patients’ distrust extends to their providers. This has important clinical implications, as a lack of trust between patients and clinicians is associated with reduced treatment adherence and care-seeking behaviors [37].

In contrast, clinicians approached technology trust differently, primarily based on evidence. They expressed concern that patients might over-rely on technology rather than trusting clinical judgment, highlighting a fundamental tension in how different stakeholders view the role of technology in care delivery.

### The Value of Hybrid Delivery

Building on the foundation of trust, our findings emphasize the importance of balancing digital and in-person care. Previous research has shown that psychological and emotional bonds are strengthened through in-person interactions, where nonverbal cues play a crucial role [120]. Our review confirms that in-person and remote consultations can best promote trust and maintain therapeutic relationships. Hybrid arrangements that offer flexibility and choice in consultation mode can help prevent depersonalization while retaining the benefits of digital care [121]. In addition, multiple recent studies have recommended that for effective implementation and to ensure high-quality telehealth care, it is essential to support implementation at each stage of the process. Organizations must provide guidelines and support for health care professionals, anticipating both technological and human challenges and their corresponding solutions [122]. Based on our findings and those from the literature, we recommend providing education and training to support individuals with access to technology effectively, as well as advocating for those who do not have access to these technologies. This could prevent disadvantaged groups from being excluded further. Although we found no trial evidence for specific interventions, it is widely recommended that training not only focus on professional development [123] but also supports to benefit from digital health care [124].

### Implementation of Technology

Perceived threats to patient and health care professional relationships have been identified as one of the main barriers to implementing technology [125]. It is thus disappointing that only 11 out of 97 (12%) of our included papers focused explicitly on this crucial issue. Research is needed to ensure that the future application of digital technology aligns with patients’ wishes, and their ability to cope, and build confidence and trust in health technologies [126]. Keywords and phrases include hybrid systems, flexibility, and recognizing individual preferences.

Recognizing that the perceived motivation behind using health technology influences trust in the provider and the relationship [49] enables those driving the implementation of technology to shape perceptions. For instance, Adjekum et al [126] responded to the observation that users trusted public institutions and universities more than profit-making entities.

Multiple contextual factors may influence whether and how digital health care is adopted, as well as its impact on the patient-professional relationship. Time and resource constraints within health care systems already negatively impact patient-professional relationships [127]. Improved communication and efficiency benefits of technology may mitigate these effects—or, conversely, exacerbate them if digital health care places additional demands on services or raises unrealistic expectations. Patient demographics such as gender and age [128] may affect preferences for delivery of care, so the introduction of digital health care will have a different impact on the underlying patient and professional relationship; disease type and severity will further affect the potential of technology to a patient’s ability to interact and engage with health care professionals.

### Strengths and Limitations

A key strength of our review was the robust methodology. We followed established systematic review procedures, including duplicate screening and data extraction, supported by a comprehensive search strategy developed in collaboration with a medical librarian. Our novel “crowdsourcing” approach, involving trained volunteers, enabled the timely completion of tasks while maintaining quality through extensive training, careful oversight, and verification of all data extraction by senior reviewers (MS and DD). Another significant strength was the involvement of stakeholders from the CONNECT and DRAGON projects in the design and interpretation of findings. The stakeholder workshop enabled us to “sense-check” our analysis, helping us refine the synthesis and interpretation, as reflected in the schema, which underwent significant revision following the workshop. Using the CASP checklist is a strength because it focuses on the applicability of studies to answer our research question, highlighting that only a minority of the studies had an explicit aim related to exploring the patient-professional relationship. CASP requests an assessment of “validity” but does not provide a summary score for the risk of bias. Whilst this is a limitation of this tool, we were not planning to assess effect size given the highly heterogeneous contexts and secondary outcome measures.

However, several limitations should be noted. We may have missed relevant papers despite screening over 15,000 titles and abstracts. A fundamental limitation was that most included studies were designed to test the efficacy and safety of technology rather than exploring the patient-professional relationship. Few papers explicitly addressed our aim as their primary objective, with most reporting incidental findings about the patient-provider relationship; however, the findings were notably consistent across studies. Our workshop was likely to have attracted participants interested in digital technology and comfortable with the online format. Exploring the perspectives of individuals who are less confident with digital tools is a research priority.

### Implications

Recommendations for maintaining a positive patient-provider relationship using digital health technology in respiratory health care are summarized in Textbox 2.

**Recommendations for maintaining a positive patient-provider relationship using digital health technology.**

**Recommendations**

- Be transparent about implementation motivations.
- Ensure technology performance is reliable.
- Account for individual circumstances and access.
- Offer hybrid care combining remote and in-person consultations.
- Consider establishing initial relationships in person.
- Maintain continuity of care with the same health care provider.
- Offer hybrid care combining remote and in-person consultations.
- Support access to self-monitoring tools and health information.

**Rationale**

- Trust was enhanced when digital health care was used to increase patient safety and convenience but diminished if technology was perceived as primarily cost-saving.
- Poor functionality led to loss of confidence and negatively impacted patient-provider relationships, with evidence of missing data from monitoring devices leading to loss of trust.
- Socioeconomic factors affected confidence and trust in technology, with some populations (older adults and socioeconomically deprived) finding access challenging.
- Both patients and providers reported depersonalization with remote-only care.
- Both clinicians and patients found remote interactions with new providers difficult and highlighted challenges in establishing trust and rapport.
- Patients were more trusting when the same provider consistently performed telemonitoring.
- Both patients and providers reported depersonalization with remote-only care.
- Technology helped patients be better informed about their condition and increased their self-efficacy through home-collected data, enabling active participation in self-management.

### Conclusion

Digital health technology impacts trust in respiratory care through four key mechanisms: adoption factors, confidence in technology, connection, and patient empowerment. Our findings emphasize that successful integration of digital health technology in respiratory care requires transparent implementation motivations, consideration of individual patient circumstances, and maintenance of human connection—all underpinned by reliable technology that supports rather than replaces the therapeutic relationship.

## Appendix

Multimedia Appendix 1 Search strategy.

Multimedia Appendix 2 PRISMA checklist.

Multimedia Appendix 3 Quantitative findings.

## Abbreviations

AI: artificial intelligence
CASP: Critical Appraisal Skills Programme
DRAGON: The Rapid and Secure AI-enhanced Diagnosis, Precision Medicine and Patient Empowerment Centered Decision Support System for Coronavirus Pandemics
COPD: chronic obstructive pulmonary disease
PRISMA: Preferred Reporting Items for Systematic Reviews and Meta-Analyses
PROSPERO: International Prospective Register of Systematic Reviews
WHO: World Health Organization
