# Supplementary material for: Impact of Digital Health on Patient-Provider Relationships in Respiratory Secondary Care Based on Qualitative and Quantitative Evidence: Systematic Review
Source: J Med Internet Res. 2025 May 30;27:e70970. doi: 10.2196/70970 (PMC12166327; doi:10.2196/70970)
Supplement: Multimedia Appendix 1 [file jmir_v27i1e70970_app1.docx]

**Search Strategy**

A broad search was developed combining terms for the respiratory conditions of interest with terms relating to telehealth and terms relating to the patient-provider relationship, including terms of known measures to assess this. The setting of secondary care was not included in the search string, as following scoping work, this was found to be too narrow and missed studies of potential interest. Free-text searches and subject heading searches were used (where available). No limits were applied to the search. Searches were conducted in November 2023 on the following databases: Ovid MEDLINE, Embase via Ovid, CINAHL via ESBCO, Cochrane Database of Systematic Reviews, Cochrane Central Register of Controlled Trials, PsycINFO via Ovid. The full search strategies can be found in Appendix 1.

**Appendix 1: Search strategies**

Ovid MEDLINE(R) and Epub Ahead of Print, In-Process, In-Data-Review & Other Non-Indexed Citations, Daily and Versions <1946 to November 07, 2023>

Search Conducted 8 November 2023

1 exp Asthma/ 143492

2 asthma*.ti,ab. 180371

3 exp Bronchiectasis/ 10341

4 bronchiectas*.ti,ab. 11748

5 exp Pulmonary Disease, Chronic Obstructive/ 67777

6 (chronic airflow obstruction* or COAD or COPD).ti,ab. 59564

7 (chronic obstructive adj (airway disease* or lung disease* or pulmonary disease*)).ti,ab. 65099

8 exp Cystic Fibrosis/ 40216

9 cystic fibrosis.ti,ab. 50494

10 exp Lung Neoplasms/ 278139

11 ((lung or pulmonary) adj2 (cancer* or neoplasm*)).ti,ab. 212031

12 exp Sleep Apnea, Obstructive/ 27580

13 sleep apnea*.ti,ab. 39254

14 osahs.ti,ab. 1703

15 exp Pneumonia/ 346552

16 (pneumonia* or pneumoniti*).ti,ab. 223793

17 ((lung or pulmonary) adj inflammation*).ti,ab. 13647

18 exp Pulmonary Fibrosis/ 28489

19 (pulmonary fibrosis or fibrosing alveoliti*).ti,ab. 24759

20 pulmonary vascular disease.ti,ab. 1787

21 exp Tuberculosis/ 207341

22 (tuberculos* or TB or koch* disease).ti,ab. 243381

23 exp alpha 1-Antitrypsin Deficiency/ 3759

24 ((alpha 1 or alpha-1) adj antitrypsin deficienc*).ti,ab. 2775

25 exp Influenza, Human/ 58416

26 (influenza* or flu or grippe).ti,ab. 146882

27 exp Pneumonia, Viral/ or exp COVID-19/ or exp Coronavirus Infections/ or exp SARS-CoV-2/ 268079

28 (COVID or COVID-19 or COVID19 or "SARS‐CoV‐2" or "SARS‐CoV2" or SARSCoV2 or "SARSCoV‐2" or "SARS coronavirus 2" or "2019 nCoV" or "2019nCoV" or "2019‐novel CoV" or "nCov 2019" or "nCov 19" or coronavirus* or corona-virus* or corona).ti,ab. 380625

29 exp Respiratory Tract Diseases/ 1747090

30 exp Lung Diseases/ 1244653

31 (respiratory adj1 disease*).ti,ab. 40727

32 lung disease*.ti,ab. 66164

33 pulmonary disease*.ti,ab. 82609

34 or/1-33 2460326

35 exp Telecommunications/ 125956

36 exp Culturally Appropriate Technology/ 19

37 exp Information Technology/ 837

38 ((communicat* or health* or informat* or comput* or medical or integrat* or digital or online) adj3 (technol* or applicat*)).ti,ab. 129190

39 ((informat* or communicat*) adj3 (exchang* or tech*)).ti,ab. 47154

40 (ehealth or e-health or electronic health or telehealth or tele-health or telemedicine or tele-medicine or tele-nursing or telenursing or telecommunicat* or tele-communicat* or videoconferenc* or video-conferenc* or video conferenc* or virtual care or teleradio* or teleradio* or telemetry or mobile app* or smartphone* or mobile health or mhealth or m-health or software).ti,ab. 354445

41 ((virtual or remote* or distance or distant or online or mobile or video or asynchronous or digital) adj3 (consult* or health or healthcare or medicine)).ti,ab. 32384

42 exp Telemedicine/ 45724

43 (iPad or iPhone or handheld device* or hand held device* or hand-held device* or mobile device* or mobile phone* or cell phone* or cellphone* or internet-based or internet based or web-based or web based or sms or short message service or android or text messag* or tablet computer*).ti,ab. 91755

44 exp Computers, Handheld/ or exp Mobile Applications/ or exp Software/ or exp Cell Phone/ 205379

45 exp Smartphone/ 9402

46 exp Internet/ 98642

47 exp Remote Consultation/ 5853

48 or/35-47 803829

49 34 and 48 63929

50 exp Professional-Patient Relations/ 148675

51 exp Physician-Patient Relations/ 76410

52 exp Interprofessional Relations/ 72666

53 ((Patient* or client*) adj3 (provider* or physician* or clinician* or health professional*)).ti,ab. 125123

54 ((doctor* or physician* or provider* or health professional* or clinician*) adj3 (relation* or communicat* or trust or loyal* or empath* or knowledge* or scale or subscale or sub-scale or questionnaire or assessment or inventory or measure or measurement)).ti,ab. 70011

55 ("provider-provider relation*" or "team relation*" or "inter?professional relation*" or "inter?clinician relation*" or "care team relation*" or "interprofessional collaboration*").ti,ab. 3344

56 SEGUE Framework.ti,ab. 15

57 Jefferson Scale of Empathy.ti,ab. 297

58 (Difficult Doctor-Patient Relationship Questionnaire or DDPRQ-10).ti,ab. 27

59 "Consultation and Relational Empathy Measure".ti,ab. 38

60 Patient-Doctor Relationship Questionnaire.ti,ab. 42

61 exp Empathy/ 23091

62 exp Trust/ 13153

63 *Communication/ 42521

64 "Patient Doctor Depth of Relationship".ti,ab. 8

65 Human Connection scale.ti,ab. 11

66 ("4-Point Alliance Scale" or 4-PAS).ti,ab. 27

67 Kim Alliance Scale.ti,ab. 6

68 Dual-Role Relationships Inventory.ti,ab. 2

69 Inpatient-Treatment Alliance Scale.ti,ab. 3

70 "Stanford Trust in Physician scale".ti,ab. 0

71 Working Alliance Inventory.ti,ab. 380

72 (Helping Alliance questionnaire or Helping Alliance questionnaire-revised or HAq-R).ti,ab. 132

73 Agnew Relationship Measure.ti,ab. 12

74 (California Psychotherapy Alliance Scales or CALPAS).ti,ab. 23

75 patient-physician relationship questionnaire.ti,ab. 0

76 ("relational communication scale for observers" or RCS-O).ti,ab. 4

77 (Wake Forest adj1 Trust Scale).ti,ab. 6

78 Health Care Relationship Trust Scale.ti,ab. 3

79 (("Consultation and Relational Empathy" or CARE) adj measure).ti,ab. 425

80 (Vanderbilt Therapeutic Alliance Scale or VTAS).ti,ab. 151

81 "Scale To Assess therapeutic Relationship".ti,ab. 4

82 (doctor-patient relationship scale or DPRS).ti,ab. 257

83 Set Elicit Give Understand End.ti,ab. 1

84 "Physician Trust in the Patient Scale".ti,ab. 0

85 "Interpersonal Process of Care".ti,ab. 8

86 PDRQ-9.ti,ab. 32

87 Patient-Practitioner Orientation Scale.ti,ab. 94

88 ("Clinical and Group Consumer Assessment of Healthcare Providers and Systems" or CG-CAHPS).ti,ab. 60

89 Nijmegen continuity questionnaire.ti,ab. 16

90 therapeutic bond scale.ti,ab. 2

91 medical care questionnaire.ti,ab. 6

92 or/50-91 417969

93 49 and 92 3820

Embase <1974 to 2023 Week 45>

Search Conducted 13 November 2023

1 *asthma/ 152611

2 asthma*.ti,ab. 263128

3 *bronchiectasis/ 7472

4 bronchiectas*.ti,ab. 19450

5 *chronic obstructive lung disease/ 83507

6 (chronic airflow obstruction* or COAD or COPD).ti,ab. 112189

7 (chronic obstructive adj (airway disease* or lung disease* or pulmonary disease*)).ti,ab. 94603

8 *cystic fibrosis/ 54709

9 cystic fibrosis.ti,ab. 78376

10 *lung tumor/ 39559

11 ((lung or pulmonary) adj2 (cancer* or neoplasm*)).ti,ab. 321652

12 *sleep disordered breathing/ 33896

13 sleep apnea*.ti,ab. 65588

14 osahs.ti,ab. 2341

15 *pneumonia/ 50656

16 (pneumonia* or pneumoniti*).ti,ab. 319898

17 ((lung or pulmonary) adj inflammation*).ti,ab. 19714

18 *lung fibrosis/ 17645

19 (pulmonary fibrosis or fibrosing alveoliti*).ti,ab. 39093

20 pulmonary vascular disease.ti,ab. 3118

21 *tuberculosis/ 73571

22 (tuberculos* or TB or koch* disease).ti,ab. 242828

23 *alpha 1 antitrypsin deficiency/ 3502

24 ((alpha 1 or alpha-1) adj antitrypsin deficienc*).ti,ab. 3420

25 *influenza/ 34223

26 (influenza* or flu or grippe).ti,ab. 172279

27 *virus pneumonia/ 6483

28 *coronavirus disease 2019/ 282107

29 *Coronavirus infection/ 4694

30 *Severe acute respiratory syndrome coronavirus 2/ 40710

31 (COVID or COVID-19 or COVID19 or "SARS‐CoV‐2" or "SARS‐CoV2" or SARSCoV2 or "SARSCoV‐2" or "SARS coronavirus 2" or "2019 nCoV" or "2019nCoV" or "2019‐novel CoV" or "nCov 2019" or "nCov 19" or coronavirus* or corona-virus* or corona).ti,ab. 428892

32 *respiratory tract disease/ 21506

33 *lung disease/ 34650

34 (respiratory adj1 disease*).ti,ab. 54826

35 lung disease*.ti,ab. 107599

36 pulmonary disease*.ti,ab. 120331

37 or/1-36 2144984

38 *telecommunication/ 10518

39 *appropriate technology/ 31

40 *information technology/ 3432

41 ((communicat* or health* or informat* or comput* or medical or integrat* or digital or online) adj3 (technol* or applicat*)).ti,ab. 154534

42 ((informat* or communicat*) adj3 (exchang* or tech*)).ti,ab. 56623

43 (ehealth or e-health or electronic health or telehealth or tele-health or telemedicine or tele-medicine or tele-nursing or telenursing or telecommunicat* or tele-communicat* or videoconferenc* or video-conferenc* or video conferenc* or virtual care or teleradio* or teleradio* or telemetry or mobile app* or smartphone* or mobile health or mhealth or m-health or software).ti,ab. 546977

44 ((virtual or remote* or distance or distant or online or mobile or video or asynchronous or digital) adj3 (consult* or health or healthcare or medicine)).ti,ab. 38595

45 *telemedicine/ 24020

46 (iPad or iPhone or handheld device* or hand held device* or hand-held device* or mobile device* or mobile phone* or cell phone* or cellphone* or internet-based or internet based or web-based or web based or sms or short message service or android or text messag* or tablet computer*).ti,ab. 123828

47 *personal digital assistant/ 655

48 *mobile application/ 11046

49 *software/ 15696

50 *mobile phone/ 8525

51 *smartphone/ 9208

52 *Internet/ 38119

53 *teleconsultation/ 4602

54 or/38-53 862760

55 37 and 54 71452

56 *professional-patient relationship/ 1768

57 *doctor patient relationship/ 2328

58 *public relations/ 28373

59 ((Patient* or client*) adj3 (provider* or physician* or clinician* or health professional*)).ti,ab. 186810

60 ((doctor* or physician* or provider* or health professional* or clinician*) adj3 (relation* or communicat* or trust or loyal* or empath* or knowledge* or scale or subscale or sub-scale or questionnaire or assessment or inventory or measure or measurement)).ti,ab. 100729

61 ("provider-provider relation*" or "team relation*" or "inter?professional relation*" or "inter?clinician relation*" or "care team relation*" or "interprofessional collaboration*").ti,ab. 3990

62 SEGUE Framework.ti,ab. 16

63 Jefferson Scale of Empathy.ti,ab. 303

64 (Difficult Doctor-Patient Relationship Questionnaire or DDPRQ-10).ti,ab. 31

65 "Consultation and Relational Empathy Measure".ti,ab. 43

66 Patient-Doctor Relationship Questionnaire.ti,ab. 51

67 *empathy/ 12444

68 *trust/ 7585

69 *interpersonal communication/ 51153

70 "Patient Doctor Depth of Relationship".ti,ab. 12

71 Human Connection scale.ti,ab. 17

72 ("4-Point Alliance Scale" or 4-PAS).ti,ab. 60

73 Kim Alliance Scale.ti,ab. 11

74 Dual-Role Relationships Inventory.ti,ab. 2

75 Inpatient-Treatment Alliance Scale.ti,ab. 6

76 "Stanford Trust in Physician scale".ti,ab. 0

77 Working Alliance Inventory.ti,ab. 468

78 (Helping Alliance questionnaire or Helping Alliance questionnaire-revised or HAq-R).ti,ab. 219

79 Agnew Relationship Measure.ti,ab. 13

80 (California Psychotherapy Alliance Scales or CALPAS).ti,ab. 34

81 patient-physician relationship questionnaire.ti,ab. 0

82 ("relational communication scale for observers" or RCS-O).ti,ab. 5

83 (Wake Forest adj1 Trust Scale).ti,ab. 8

84 Health Care Relationship Trust Scale.ti,ab. 4

85 (("Consultation and Relational Empathy" or CARE) adj measure).ti,ab. 590

86 (Vanderbilt Therapeutic Alliance Scale or VTAS).ti,ab. 273

87 "Scale To Assess therapeutic Relationship".ti,ab. 4

88 (doctor-patient relationship scale or DPRS).ti,ab. 364

89 Set Elicit Give Understand End.ti,ab. 1

90 "Physician Trust in the Patient Scale".ti,ab. 0

91 "Interpersonal Process of Care".ti,ab. 16

92 PDRQ-9.ti,ab. 39

93 Patient-Practitioner Orientation Scale.ti,ab. 97

94 ("Clinical and Group Consumer Assessment of Healthcare Providers and Systems" or CG-CAHPS).ti,ab. 79

95 Nijmegen continuity questionnaire.ti,ab. 16

96 therapeutic bond scale.ti,ab. 3

97 medical care questionnaire.ti,ab. 7

98 or/56-97 353984

99 55 and 98 4474

CINAHL via EBSCO

Search Conducted 17 November 2023

| S98 | S54 AND S97 |
| --- | --- |
| S97 | S55 OR S56 OR S57 OR S58 OR S59 OR S60 OR S61 OR S62 OR S63 OR S64 OR S65 OR S66 OR S67 OR S68 OR S69 OR S70 OR S71 OR S72 OR S73 OR S74 OR S75 OR S76 OR S77 OR S78 OR S79 OR S80 OR S81 OR S82 OR S83 OR S84 OR S85 OR S86 OR S87 OR S88 OR S89 OR S90 OR S91 OR S92 OR S93 OR S94 OR S95 OR S96 |
| S96 | TI medical care questionnaire OR AB medical care questionnaire |
| S95 | TI therapeutic bond scale OR AB therapeutic bond scale |
| S94 | TI Nijmegen continuity questionnaire OR AB Nijmegen continuity questionnaire |
| S93 | TI ( ("Clinical and Group Consumer Assessment of Healthcare Providers and Systems" or CG-CAHPS) ) OR AB ( ("Clinical and Group Consumer Assessment of Healthcare Providers and Systems" or CG-CAHPS) ) |
| S92 | TI Patient-Practitioner Orientation Scale OR AB Patient-Practitioner Orientation Scale |
| S91 | TI PDRQ-9 OR AB PDRQ-9 |
| S90 | TI "Interpersonal Process of Care" OR AB "Interpersonal Process of Care" |
| S89 | TI "Physician Trust in the Patient Scale" OR AB "Physician Trust in the Patient Scale" |
| S88 | TI Set Elicit Give Understand End OR AB Set Elicit Give Understand End |
| S87 | TI ( (doctor-patient relationship scale or DPRS) ) OR AB ( (doctor-patient relationship scale or DPRS) ) |
| S86 | TI "Scale To Assess therapeutic Relationship" OR AB "Scale To Assess therapeutic Relationship" |
| S85 | TI ( (Vanderbilt Therapeutic Alliance Scale or VTAS) ) OR AB ( (Vanderbilt Therapeutic Alliance Scale or VTAS) ) |
| S84 | TI ( (("Consultation and Relational Empathy" N1 measure) ) OR AB ( (("Consultation and Relational Empathy") N1 measure) ) |
| S83 | TI Health Care Relationship Trust Scale OR AB Health Care Relationship Trust Scale |
| S82 | TI (Wake Forest N1 Trust Scale) OR AB (Wake Forest N1 Trust Scale) |
| S81 | TI ( ("relational communication scale for observers" or RCS-O) ) OR AB ( ("relational communication scale for observers" or RCS-O) ) |
| S80 | TI patient-physician relationship questionnaire OR AB patient-physician relationship questionnaire |
| S79 | TI ( (California Psychotherapy Alliance Scales or CALPAS) ) OR AB ( (California Psychotherapy Alliance Scales or CALPAS) ) |
| S78 | TI Agnew Relationship Measure OR AB Agnew Relationship Measure |
| S77 | TI ( (Helping Alliance questionnaire or Helping Alliance questionnaire-revised or HAq-R) ) OR AB ( (Helping Alliance questionnaire or Helping Alliance questionnaire-revised or HAq-R) ) |
| S76 | TI Working Alliance Inventory OR AB Working Alliance Inventory |
| S75 | TI "Stanford Trust in Physician scale" OR AB "Stanford Trust in Physician scale" |
| S74 | TI Inpatient-Treatment Alliance Scale OR AB Inpatient-Treatment Alliance Scale |
| S73 | TI Dual-Role Relationships Inventory OR AB Dual-Role Relationships Inventory |
| S72 | TI Kim Alliance Scale OR AB Kim Alliance Scale |
| S71 | TI ( ("4-Point Alliance Scale" or 4-PAS) ) OR AB ( ("4-Point Alliance Scale" or 4-PAS) ) |
| S70 | TI Human Connection scale OR AB Human Connection scale |
| S69 | TI "Patient Doctor Depth of Relationship" OR AB "Patient Doctor Depth of Relationship" |
| S68 | (MM "Communication") |
| S67 | (MH "Trust") |
| S66 | (MH "Empathy") |
| S65 | TI Patient-Doctor Relationship Questionnaire OR AB Patient-Doctor Relationship Questionnaire |
| S64 | TI ( "Consultation and Relational Empathy Measure" ) OR AB ( "Consultation and Relational Empathy Measure" ) |
| S63 | TI ( (Difficult Doctor-Patient Relationship Questionnaire or DDPRQ-10) ) OR AB ( (Difficult Doctor-Patient Relationship Questionnaire or DDPRQ-10) ) |
| S62 | TI Jefferson Scale of Empathy OR AB Jefferson Scale of Empathy |
| S61 | TI SEGUE Framework OR AB SEGUE Framework |
| S60 | TI ( ("provider-provider relation*" or "team relation*" or "inter?professional relation*" or "inter?clinician relation*" or "care team relation" or "interprofessional collaboration*") ) OR AB ( ("provider-provider relation*" or "team relation*" or "inter?professional relation*" or "inter?clinician relation*" or "care team relation" or "interprofessional collaboration*") ) |
| S59 | TI ( ((doctor* or physician* or provider* or health professional* or clinician*) N3 (relation* or communicat* or trust or loyal* or empath* or knowledge* or scale or subscale or sub-scale or questionnaire or assessment or inventory or measure or measurement)) ) |
| S58 | TI ( ((Patient* or client*) N3 (provider* or physician* or clinician* or health professional*)) ) |
| S57 | (MH "Interprofessional Relations+") |
| S56 | (MH "Physician-Patient Relations") |
| S55 | (MH "Professional-Patient Relations+") |
| S54 | S37 AND S53 |
| S53 | S37 OR S38 OR S39 OR S40 OR S41 OR S42 OR S43 OR S44 OR S45 OR S46 OR S47 OR S48 OR S49 OR S50 OR S51 OR S52 |
| S52 | (MH "Remote Consultation") |
| S51 | (MH "Internet+") |
| S50 | (MH "Smartphone") |
| S49 | (MH "Cellular Phone+") |
| S48 | (MH "Software+") |
| S47 | (MH "Mobile Applications") |
| S46 | (MH "Computers, Hand-Held+") |
| S45 | TI ( (iPad or iPhone or handheld device* or hand held device* or hand-held device* or mobile device* or mobile phone* or cell phone* or cellphone* or internet-based or internet based or web-based or web based or sms or short message service or android or text messag* or tablet computer*) ) |
| S44 | (MH "Telemedicine+") |
| S43 | TI ( ((virtual or remote* or distance or distant or online or mobile or video or asynchronous or digital) N3 (consult* or health or healthcare or medicine)) ) |
| S42 | TI ( (ehealth or e-health or electronic health or telehealth or tele-health or telemedicine or tele-medicine or tele-nursing or telenursing or telecommunicat* or tele-communicat* or videoconferenc* or video-conferenc* or video conferenc* or virtual care or teleradio* or teleradio* or telemetry or mobile app* or smartphone* or mobile health or mhealth or m-health or software) ) |
| S41 | TI ( ((informat* or communicat*) N3 (exchang* or tech*)) ) |
| S40 | TI ( ((communicat* or health* or informat* or comput* or medical or integrat* or digital or online) N3 (technol* or applicat*)) ) |
| S39 | (MH "Information Technology+") |
| S38 | (MH "Telecommunications+") |
| S37 | S1 OR S2 OR S3 OR S4 OR S5 OR S6 OR S7 OR S8 OR S9 OR S10 OR S11 OR S12 OR S13 OR S14 OR S15 OR S16 OR S17 OR S18 OR S19 OR S20 OR S21 OR S22 OR S23 OR S24 OR S25 OR S26 OR S27 OR S28 OR S29 OR S30 OR S31 OR S32 OR S33 OR S34 OR S35 OR S36 |
| S36 | TI pulmonary disease* |
| S35 | TI lung disease* |
| S34 | TI (respiratory N1 disease*) |
| S33 | (MH "Lung Diseases+") |
| S32 | (MH "Respiratory Tract Diseases+") |
| S31 | TI ( (COVID or COVID-19 or COVID19 or "SARS‐CoV‐2" or "SARS‐CoV2" or SARSCoV2 or "SARSCoV‐2" or "SARS coronavirus 2" or "2019 nCoV" or "2019nCoV" or "2019‐novel CoV" or "nCov 2019" or "nCov 19" or coronavirus* or corona-virus* or corona) ) |
| S30 | (MH "SARS-CoV-2") |
| S29 | (MH "Coronavirus Infections+") |
| S28 | (MH "COVID-19+") |
| S27 | (MH "Pneumonia, Viral") |
| S26 | TI ( (influenza* or flu or grippe) ) |
| S25 | (MH "Influenza, Human+") |
| S24 | TI ( ((alpha 1 or alpha-1) N1 antitrypsin deficienc*) ) |
| S23 | (MH "Alpha 1-Antitrypsin Deficiency") |
| S22 | TI ( (tuberculos* or TB or koch* disease) ) |
| S21 | (MH "Tuberculosis+") |
| S20 | TI pulmonary vascular disease |
| S19 | TI ( (pulmonary fibrosis or fibrosing alveoliti*) ) |
| S18 | (MH "Pulmonary Fibrosis+") |
| S17 | TI ( ((lung or pulmonary) N1 inflammation*) ) |
| S16 | TI ( (pneumonia* or pneumoniti*) ) |
| S15 | (MH "Pneumonia+") |
| S14 | TI osahs |
| S13 | TI sleep apnea* |
| S12 | (MH "Sleep Apnea, Obstructive") |
| S11 | TI ((lung or pulmonary) N2 (cancer* or neoplasm*)) |
| S10 | (MH "Lung Neoplasms+") |
| S9 | TI cystic fibrosis OR AB cystic fibrosis |
| S8 | (MH "Cystic Fibrosis") |
| S7 | TI ( (chronic obstructive N1 (airway disease* or lung disease* or pulmonary disease*)) ) |
| S6 | TI ( (chronic airflow obstruction* or COAD or COPD) ) |
| S5 | (MH "Pulmonary Disease, Chronic Obstructive+") |
| S4 | TI bronchiectas* |
| S3 | (MH "Bronchiectasis") |
| S2 | TI asthma* |
| S1 | (MH "Asthma+") |

Cochrane Library (CDSR & Central)

Search Conducted 20 November 2023

#1 MeSH descriptor: [Asthma] explode all trees 15057

#2 asthma* 40142

#3 MeSH descriptor: [Bronchiectasis] explode all trees 547

#4 bronchiectas* 1646

#5 MeSH descriptor: [Pulmonary Disease, Chronic Obstructive] explode all trees 7317

#6 (chronic airflow obstruction* or COAD or COPD) 19852

#7 (chronic obstructive NEXT (airway disease* or lung disease* or pulmonary disease*)) 18416

#8 MeSH descriptor: [Cystic Fibrosis] explode all trees 2535

#9 cystic fibrosis 6860

#10 MeSH descriptor: [Lung Neoplasms] explode all trees 10583

#11 ((lung or pulmonary) NEAR/2 (cancer* or neoplasm*)) 26826

#12 MeSH descriptor: [Sleep Apnea, Obstructive] explode all trees 2715

#13 sleep apnea* 8087

#14 osahs 274

#15 MeSH descriptor: [Pneumonia] explode all trees 10637

#16 (pneumonia* or pneumoniti*) 24863

#17 ((lung or pulmonary) adj inflammation*) 246

#18 MeSH descriptor: [Pulmonary Fibrosis] explode all trees 791

#19 (pulmonary fibrosis or fibrosing alveoliti*) 4831

#20 pulmonary vascular disease 2752

#21 MeSH descriptor: [Tuberculosis] explode all trees 3333

#22 (tuberculos* or TB or koch* disease) 14986

#23 MeSH descriptor: [alpha 1-Antitrypsin Deficiency] explode all trees 100

#24 ((alpha 1 or alpha-1) NEXT antitrypsin deficienc*) 236

#25 MeSH descriptor: [Influenza, Human] explode all trees 3291

#26 (influenza* or flu or grippe) 13532

#27 MeSH descriptor: [Pneumonia, Viral] explode all trees 5008

#28 MeSH descriptor: [COVID-19] explode all trees 4894

#29 MeSH descriptor: [Coronavirus Infections] explode all trees 5487

#30 MeSH descriptor: [SARS-CoV-2] explode all trees 2419

#31 (COVID or COVID-19 or COVID19 or "SARS‐CoV‐2" or "SARS‐CoV2" or SARSCoV2 or "SARSCoV‐2" or "SARS coronavirus 2" or "2019 nCoV" or "2019nCoV" or "2019‐novel CoV" or "nCov 2019" or "nCov 19" or coronavirus* or corona-virus* or corona) 19435

#32 MeSH descriptor: [Respiratory Tract Diseases] explode all trees 86418

#33 MeSH descriptor: [Lung Diseases] explode all trees 59081

#34 (respiratory NEAR/1 disease*) 6017

#35 lung disease* 48551

#36 pulmonary disease* 37800

#37 {OR #1-#36} 209107

#38 MeSH descriptor: [Telecommunications] explode all trees 10933

#39 MeSH descriptor: [Culturally Appropriate Technology] explode all trees 0

#40 MeSH descriptor: [Information Technology] explode all trees 42

#41 ((communicat* or health* or informat* or comput* or medical or integrat* or digital or online) NEAR/3 (technol* or applicat*)) 14832

#42 ((informat* or communicat*) NEAR/3 (exchang* or tech*)) 3638

#43 (ehealth or e-health or electronic health or telehealth or tele-health or telemedicine or tele-medicine or tele-nursing or telenursing or telecommunicat* or tele-communicat* or videoconferenc* or video-conferenc* or video conferenc* or virtual care or teleradio* or teleradio* or telemetry or mobile app* or smartphone* or mobile health or mhealth or m-health or software) 87547

#44 ((virtual or remote* or distance or distant or online or mobile or video or asynchronous or digital) NEAR/3 (consult* or health or healthcare or medicine)) 8957

#45 MeSH descriptor: [Telemedicine] explode all trees 4313

#46 (iPad or iPhone or handheld device* or hand held device* or hand-held device* or mobile device* or mobile phone* or cell phone* or cellphone* or internet-based or internet based or web-based or web based or sms or short message service or android or text messag* or tablet computer*) 55309

#47 MeSH descriptor: [Computers, Handheld] explode all trees 1390

#48 MeSH descriptor: [Mobile Applications] explode all trees 1607

#49 MeSH descriptor: [Software] explode all trees 6403

#50 MeSH descriptor: [Cell Phone] explode all trees 3173

#51 MeSH descriptor: [Smartphone] explode all trees 1042

#52 MeSH descriptor: [Internet] explode all trees 6250

#53 MeSH descriptor: [Remote Consultation] explode all trees 442

#54 {OR #37-#53} 326398

#55 #36 AND #54 37800

#56 MeSH descriptor: [Professional-Patient Relations] explode all trees 3251

#57 MeSH descriptor: [Physician-Patient Relations] explode all trees 1742

#58 MeSH descriptor: [Interprofessional Relations] explode all trees 730

#59 ((Patient* or client*) NEAR/3 (provider* or physician* or clinician* or health professional*)) 53133

#60 ((doctor* or physician* or provider* or health professional* or clinician*) NEAR/3 (relation* or communicat* or trust or loyal* or empath* or knowledge* or scale or subscale or sub-scale or questionnaire or assessment or inventory or measure or measurement)) 75555

#61 (provider-provider relation* or team relation* or inter?professional relation* or inter?clinician relation* or care team relation* or interprofessional collaboration*) 6054

#62 SEGUE Framework 1

#63 Jefferson Scale of Empathy 85

#64 (Difficult Doctor-Patient Relationship Questionnaire or DDPRQ-10) 62

#65 "Consultation and Relational Empathy Measure" 10

#66 Patient-Doctor Relationship Questionnaire 37

#67 MeSH descriptor: [Empathy] explode all trees 833

#68 MeSH descriptor: [Trust] explode all trees 430

#69 MeSH descriptor: [Communication] this term only 3155

#70 "Patient Doctor Depth of Relationship" 3

#71 Human Connection scale 435

#72 (4 Point Alliance Scale or 4 PAS) 1964

#73 Kim Alliance Scale 56

#74 Dual-Role Relationships Inventory 6

#75 Inpatient-Treatment Alliance Scale 20

#76 "Stanford Trust in Physician scale" 0

#77 Working Alliance Inventory 400

#78 (Helping Alliance questionnaire or Helping Alliance questionnaire-revised or HAq-R) 173

#79 Agnew Relationship Measure 15

#80 (California Psychotherapy Alliance Scales or CALPAS) 26

#81 patient-physician relationship questionnaire 63

#82 ("relational communication scale for observers" or RCS-O) 1

#83 (Wake Forest NEAR/1 Trust Scale) 2

#84 Health Care Relationship Trust Scale 624

#85 (("Consultation and Relational Empathy" or CARE) NEXT measure) 116

#86 (Vanderbilt Therapeutic Alliance Scale or VTAS) 29

#87 "Scale To Assess therapeutic Relationship" 2

#88 (doctor-patient relationship scale or DPRS) 595

#89 Set Elicit Give Understand End 34

#90 "Physician Trust in the Patient Scale" 0

#91 "Interpersonal Process of Care" 3

#92 PDRQ-9 2

#93 Patient-Practitioner Orientation Scale 6

#94 ("Clinical and Group Consumer Assessment of Healthcare Providers and Systems" or CG-CAHPS) 12

#95 Nijmegen continuity questionnaire 13

#96 therapeutic bond scale 203

#97 medical care questionnaire 17184

#98 {OR #56-#97} 126366

#99 #55 AND #98 4374

APA PsycInfo <1806 to November Week 2 2023>

Search Conducted 20 November 2023

1 exp Asthma/ 5327

2 asthma*.ti,ab. 8609

3 exp Bronchial Disorders/ 181

4 bronchiectas*.ti,ab. 45

5 (chronic airflow obstruction* or COAD or COPD).ti,ab. 1945

6 (chronic obstructive adj (airway disease* or lung disease* or pulmonary disease*)).ti,ab. 2653

7 exp Cystic Fibrosis/ 1010

8 cystic fibrosis.ti,ab. 1326

9 ((lung or pulmonary) adj2 (cancer* or neoplasm*)).ti,ab. 3227

10 sleep apnea*.ti,ab. 4698

11 osahs.ti,ab. 79

12 exp Pneumonia/ 775

13 (pneumonia* or pneumoniti*).ti,ab. 2590

14 ((lung or pulmonary) adj inflammation*).ti,ab. 104

15 (pulmonary fibrosis or fibrosing alveoliti*).ti,ab. 92

16 pulmonary vascular disease.ti,ab. 7

17 exp Tuberculosis/ 1392

18 (tuberculos* or TB or koch* disease).ti,ab. 3493

19 ((alpha 1 or alpha-1) adj antitrypsin deficienc*).ti,ab. 38

20 (influenza* or flu or grippe).ti,ab. 4119

21 exp Pneumonia, Viral/ or exp COVID-19/ or exp Coronavirus Infections/ or exp SARS-CoV-2/ 28657

22 (COVID or COVID-19 or COVID19 or "SARS‐CoV‐2" or "SARS‐CoV2" or SARSCoV2 or "SARSCoV‐2" or "SARS coronavirus 2" or "2019 nCoV" or "2019nCoV" or "2019‐novel CoV" or "nCov 2019" or "nCov 19" or coronavirus* or corona-virus* or corona).ti,ab. 40581

23 (respiratory adj1 disease*).ti,ab. 1164

24 lung disease*.ti,ab. 1042

25 pulmonary disease*.ti,ab. 3008

26 or/1-25 72221

27 exp Telecommunications/ 22894

28 exp Information Technology/ 207572

29 ((communicat* or health* or informat* or comput* or medical or integrat* or digital or online) adj3 (technol* or applicat*)).ti,ab. 43892

30 ((informat* or communicat*) adj3 (exchang* or tech*)).ti,ab. 27133

31 (ehealth or e-health or electronic health or telehealth or tele-health or telemedicine or tele-medicine or tele-nursing or telenursing or telecommunicat* or tele-communicat* or videoconferenc* or video-conferenc* or video conferenc* or virtual care or teleradio* or teleradio* or telemetry or mobile app* or smartphone* or mobile health or mhealth or m-health or software).ti,ab. 57568

32 ((virtual or remote* or distance or distant or online or mobile or video or asynchronous or digital) adj3 (consult* or health or healthcare or medicine)).ti,ab. 7406

33 exp Telemedicine/ 14020

34 (iPad or iPhone or handheld device* or hand held device* or hand-held device* or mobile device* or mobile phone* or cell phone* or cellphone* or internet-based or internet based or web-based or web based or sms or short message service or android or text messag* or tablet computer*).ti,ab. 36300

35 exp Computers, Handheld/ or exp Mobile Applications/ or exp Software/ or exp Cell Phone/ 9809

36 exp Internet/ 33833

37 or/27-36 291469

38 ((Patient* or client*) adj3 (provider* or physician* or clinician* or health professional*)).ti,ab. 30248

39 ((doctor* or physician* or provider* or health professional* or clinician*) adj3 (relation* or communicat* or trust or loyal* or empath* or knowledge* or scale or subscale or sub-scale or questionnaire or assessment or inventory or measure or measurement)).ti,ab. 25576

40 ("provider-provider relation*" or "team relation*" or "inter?professional relation*" or "inter?clinician relation*" or "care team relation" or "interprofessional collaboration*").ti,ab. 1500

41 SEGUE Framework.ti,ab. 3

42 Jefferson Scale of Empathy.ti,ab. 96

43 (Difficult Doctor-Patient Relationship Questionnaire or DDPRQ-10).ti,ab. 14

44 "Consultation and Relational Empathy Measure".ti,ab. 13

45 Patient-Doctor Relationship Questionnaire.ti,ab. 11

46 exp Empathy/ 17120

47 exp Trust/ 15519

48 *Communication/ 25516

49 "Patient Doctor Depth of Relationship".ti,ab. 4

50 Human Connection scale.ti,ab. 5

51 ("4-Point Alliance Scale" or 4-PAS).ti,ab. 3

52 Kim Alliance Scale.ti,ab. 6

53 Dual-Role Relationships Inventory.ti,ab. 2

54 Inpatient-Treatment Alliance Scale.ti,ab. 3

55 "Stanford Trust in Physician scale".ti,ab. 0

56 Working Alliance Inventory.ti,ab. 809

57 (Helping Alliance questionnaire or Helping Alliance questionnaire-revised or HAq-R).ti,ab. 109

58 Agnew Relationship Measure.ti,ab. 12

59 (California Psychotherapy Alliance Scales or CALPAS).ti,ab. 37

60 patient-physician relationship questionnaire.ti,ab. 0

61 ("relational communication scale for observers" or RCS-O).ti,ab. 1

62 (Wake Forest adj1 Trust Scale).ti,ab. 3

63 Health Care Relationship Trust Scale.ti,ab. 2

64 (("Consultation and Relational Empathy" or CARE) adj measure).ti,ab. 128

65 (Vanderbilt Therapeutic Alliance Scale or VTAS).ti,ab. 49

66 "Scale To Assess therapeutic Relationship".ti,ab. 4

67 (doctor-patient relationship scale or DPRS).ti,ab. 47

68 Set Elicit Give Understand End.ti,ab. 0

69 "Physician Trust in the Patient Scale".ti,ab. 1

70 "Interpersonal Process of Care".ti,ab. 2

71 PDRQ-9.ti,ab. 7

72 Patient-Practitioner Orientation Scale.ti,ab. 47

73 ("Clinical and Group Consumer Assessment of Healthcare Providers and Systems" or CG-CAHPS).ti,ab. 10

74 Nijmegen continuity questionnaire.ti,ab. 4

75 therapeutic bond scale.ti,ab. 3

76 medical care questionnaire.ti,ab. 2

77 or/38-76 105764

78 26 and 37 and 77 690
