# Supplementary material for: Impact of Digital Health on Patient-Provider Relationships in Respiratory Secondary Care Based on Qualitative and Quantitative Evidence: Systematic Review
Source: J Med Internet Res. 2025 May 30;27:e70970. doi: 10.2196/70970 (PMC12166327; doi:10.2196/70970)
Supplement: Multimedia Appendix 3 [file jmir_v27i1e70970_app3.docx]

| **Examples of Quantitative Evidence by Theme and Subtheme**  **Adoption Factors**  ***Clinical Context***  The clinical setting and patient characteristics had a significant influence on technology adoption rates. Zamith (2009) documented that the "majority of asthmatic patients took to home telemonitoring easily", while "chronic respiratory insufficiency (CRI) patients found it harder to adjust to." Among CRI patients, 32% reported that it was "challenging to learn to use the Doc@Home equipment," compared to only 5% of asthmatic patients. The easier adoption by asthma patients was reflected in their greater access to care, with 76% reporting they "felt they had greater ease of access to medical appointments during the program," as stated in the article.  ***Perception of Motivations***  Motivation for technology adoption varied based on patient characteristics and perceived value. Varkey (2008) found that 46% of patients were willing to pay for extended access to telemedicine. Zamith (2009) quantified motivation levels by condition, finding that among asthmatic patients, 43% (9 patients) were "very motivated" and 57% (12 patients) "moderately motivated," while CRI patients showed different patterns with 43% (19 patients) "strongly motivated," 41% (18 patients) "moderately motivated," 9% (4 patients) "indifferent," and 7% (3 patients) "reticent." Family support also differed significantly between groups, with 71% of asthmatic patients receiving family support compared to 55% of CRI patients.  **Confidence in Technology**  ***Technology Performance***  Technical reliability has a significant impact on user satisfaction with digital health systems. Varkey (2008) reported technical problems in 9% of telemedicine visits, though most patients could see (98%) and hear (98%) providers. Fadaizadeh (2015) found dramatic differences in satisfaction by device type: only 35.7% expressed satisfaction with spirometer devices compared to 85.7% satisfaction with mobile phones. Sonney (2018) reported that parent-child dyads found the mobile health application intuitive and easy to use, with high acceptability during usability testing. All studies noted that technical reliability was crucial for user adoption, with even minor technical difficulties potentially reducing compliance and satisfaction with telehealth interventions.  ***Inequitable Access***  Across these studies, significant digital disparities emerged. Byczkowski (2011) documented that while Medicaid insured 23.1% of families, they represented just 9.2% of initial portal users and a mere 3.8% of ongoing users. Similar disparities affected African American patients. Apter (2019) quantified the technological divide, with study participants having lower rates of computer ownership (53% vs. national 78%) and internet access (68% vs. national 77%). Hendra (2021) found that 6% of cystic fibrosis patients required audio-only telemedicine due to connectivity issues, with disadvantages disproportionately affecting low-income and non-English-speaking families. These findings consistently demonstrate that digital healthcare solutions, without effective implementation strategies, risk exacerbating existing healthcare disparities for vulnerable populations.  ***Strength of Evidence***  The evidence regarding the efficacy and reliability of telehealth presents mixed findings across studies. Bukstein (2022) documented increasing concerns about potential medical errors in telehealth following the onset of the pandemic, with this factor rising notably among reasons for unwillingness to recommend telehealth to colleagues. Al-Sharif (2021) reported specific participant concerns regarding the security of technology in mHealth applications, with only 50.9% of participants agreeing that Telehealth met their healthcare needs.  **Connection**  ***Communication***  Communication patterns revealed a complex picture across various telemedicine implementations. Pacht (1998) documented high patient satisfaction, with 93% of patients reporting comfort when asking questions to telemedicine physicians and 90% agreeing that physicians communicated effectively. However, only 60% preferred telemedicine visits equally to traditional in-person consultations. Agha (2009) found significant differences in verbal dominance, with a higher physician-to-patient talk ratio in telemedicine (1.45) versus in-person consultations (1.13), indicating more physician-centered communication during virtual visits. Patients made significantly more requests for repetition during telemedicine visits (1.64 vs. 0.38, p = .034), suggesting potential communication challenges. The distribution of utterances also differed notably, with physicians accounting for more utterances during telemedicine encounters (178±118) compared to patients (142±127), whereas in-person visits demonstrated a more balanced dynamic, with 166±80 physician and 166±88 patient utterances.  The intervention study by Sleath (2018) demonstrated that youth engagement could be significantly improved, with 40% of youth in the intervention group asking medication questions compared to only 20% in the usual care group. Similarly, youth in the intervention group were substantially more likely to ask about triggers (16.3% vs. 2.3%) and environmental control (5.4% vs. 1.2%). Sousa (2021) reported generally positive experiences with virtual communication during the COVID-19 pandemic, with 87.5% of patients feeling clinicians listened carefully to their questions or complaints, and 90.9% reporting that explanations were delivered clearly. Varghese (2023) found that 90.91% of respondents felt that the time allocated for asking questions and clarifying doubts during virtual consultations was sufficient, while 9.09% considered it inadequate. Additionally, this study found that providers in the intervention group were significantly more likely to educate patients about rescue medications (78% vs. 60.5%, p=0.0003), asthma triggers (59.2% vs. 45.9%, p=0.012), and environmental control measures (22.3% vs. 12.8%, p=0.019).  ***Continuity of Care***  Kneuertz et al. (2020) demonstrated that a mobile application enhanced perioperative continuity, with 54% of patients completing postoperative health checks (median of three surveys per patient), improving confidence in 85.2% of patients and reducing concerns in 81.5%.  ***Caring Presence***  Based on the telemonitoring studies reviewed, there is significant evidence that remote monitoring fosters a sense of care and support for patients with respiratory conditions. Zamith (2009) reported that 80% of patients with chronic respiratory insufficiency (CRI) felt more or much more supported through remote monitoring. This finding aligns with Fadaizadeh's (2015) study, in which patients rated the sense of increased support from medical staff as the highest among satisfaction factors, at 92.9%. Cingi (2015) demonstrated that healthcare providers maintained a consistent caring presence, with 92% of physicians responding to all urgent messages and submissions regarding severe health status. The study also found that 86% of communications occurred during standard business hours (8:00 AM-6:00 PM), indicating that telemonitoring enabled professional support during typical clinical hours, rather than requiring extended availability.  ***Depersonalisation***  Davis et al. (2021) found that 53% of patients and 90% of providers expressed at least moderate concern regarding the lack of physical examination during telehealth visits, with similar concerns about the lack of testing (42% of patients, 81% of providers). Al-Sharif (2021) reported that fewer than 50% of participants perceived telehealth approaches as similar to in-person interactions. According to Mustafa (2021), 25.9% of patients identified the absence of physical examination as their primary reason for preferring traditional in-person care. Nelson et al. (2022) documented that concerns about mistrust due to the inability to communicate in person were reported by 12.6% to 25% of the providers surveyed.  **Patient Empowerment**  ***Self-Efficacy***  Opipari-Arrigan et al. (2020) documented a statistically significant improvement in self-efficacy scores from baseline to the 6-month follow-up, with a meaningful effect size (Cohen's d = 0.36), after patients used an electronic health record–linked mHealth platform. Gashu et al. (2021) found improved adherence to patient-centered tuberculosis (TB) treatment in the intervention group (79.1%, 110/139) compared to the control group (66.4%, 95/143), suggesting enhanced self-efficacy in medication management. Kneuertz et al. (2020) reported that postoperative health checks via a mobile platform improved patient confidence in 85.2% (23/27) of patients. Shinoda et al. (2022) found that 58.8% of participants reported the telemedicine platform had a positive impact on managing their health. Coker et al. (2023) found no significant differences between the intervention and control groups in parent communication self-efficacy or asthma self-management knowledge at the 12-month follow-up.  ***Shared Decision-Making***  Opipari-Arrigan (2020) reported that shared decision-making at enrollment improved quality of life as measured by the St George's Respiratory Questionnaire (SGRQ) by 7-9 units (p<0.0001) compared to usual care, with 90% of participants reporting the electronic health record–linked mHealth platform as applicable and 88% noting improved visit collaboration. Sonney (2019) found that parents and children valued shared asthma management, with an unexpected benefit being that daily debriefs provided an opportunity for children to report on their asthma symptoms and status, which parents found more informative than relying solely on their observations. |
| --- |
